# Sirtuin1-p53, forkhead box O3a, p38 and post-infarct cardiac remodeling in the spontaneously diabetic Goto-Kakizaki rat

**DOI:** 10.1186/1475-2840-9-5

**Published:** 2010-01-27

**Authors:** Erik Vahtola, Marjut Louhelainen, Hanna Forstén, Saara Merasto, Johanna Raivio, Petri Kaheinen, Ville Kytö, Ilkka Tikkanen, Jouko Levijoki, Eero Mervaala

**Affiliations:** 1 Institute of Biomedicine, Pharmacology, University of Helsinki, Finland; 2 Department of Medicine, Helsinki University Hospital, Finland and Minerva Institute for Medical Research, Helsinki, Finland; 3 Orion Pharma Ltd, Espoo, Finland; 4 Department of Medicine, Turku University Hospital, Turku, Finland

## Abstract

**Background:**

Diabetes is associated with changes in myocardial stress-response pathways and is recognized as an independent risk factor for cardiac remodeling. Using spontaneously diabetic Goto Kakizaki rats as a model of type 2 DM we investigated whether post-translational modifications in the Akt - FOXO3a pathway, Sirt1 - p53 pathway and the mitogen activated protein kinase p38 regulator are involved in post-infarct cardiac remodeling

**Methods:**

Experimental myocardial infarction (MI) was induced by left anterior descending coronary artery ligation in spontaneously diabetic Goto-Kakizaki rats and non-diabetic Wistar controls. Cardiac function was studied by echocardiography. Myocardial hypertrophy, cardiomyocyte apoptosis and cardiac fibrosis were determined histologically 12 weeks post MI or Sham operation. Western blotting was used to study Caspase-3, Bax, Sirt1, acetylation of p53 and phosphorylation of p38, Akt and FOXO3a. Electrophoretic mobility shift assay was used to assess FOXO3a activity and its nuclear localization.

**Results:**

Post-infarct heart failure in diabetic GK rats was associated with pronounced cardiomyocyte hypertrophy, increased interstitial fibrosis and sustained cardiomyocyte apoptosis as compared with their non-diabetic Wistar controls. In the GK rat myocardium, Akt- and FOXO3a-phosphorylation was decreased and nuclear localization of FOXO3a was increased concomitantly with increased PTEN protein expression. Furthermore, increased Sirt1 protein expression was associated with decreased p53 acetylation, and phosphorylation of p38 was increased in diabetic rats with MI.

**Conclusions:**

Post-infarct heart failure in diabetic GK rats was associated with more pronounced cardiac hypertrophy, interstitial fibrosis and sustained cardiomyocyte apoptosis as compared to their non-diabetic controls. The present study suggests important roles for Akt-FOXO3a, Sirt1 - p53 and p38 MAPK in the regulation of post-infarct cardiac remodeling in type 2 diabetes.

## Background

Diabetes increases the risk for fatal myocardial infarction and development of heart failure [1-3]. The poor prognosis after myocardial infarction has been explained by the underlying diabetic cardiomyopathy exacerbated by other factors such as hypertension and ischemic heart disease. Several factors such as insulin resistance and enhanced tissue renin-angiotensin system activity presumably also contribute to the pathogenesis [4-6]. Earlier studies have provided evidence that diabetes is associated with enhanced susceptibility to cardiomyocyte apoptosis and fibrosis which, in turn, could interfere with the post-infarct cardiac remodeling process. Hyperglycemia causes the build-up of reactive oxygen species (ROS) and reactive nitrogen species (RNS) which in turn induces p53- and cytochrome-c mediated caspase-3-dependent apoptosis [7-10]. Accordingly, myocardial cell death in diabetes has been prevented by the use of antioxidants and caspase inhibitors indicating a causal role for apoptosis in the pathogenesis of diabetes-induced cardiomyocyte loss [11].

In our study we examined cardiomyocyte apoptosis, hypertrophy and interstitial fibrosis in spontaneously diabetic Goto-Kakizaki (GK) rats, a non-obese model of type II diabetes [12], Wistar rats were used as controls. To date there is lack of evidence concerning the involvement of stress response pathways and their role in MI-induced cardiac remodeling in diabetes. In this study we examined the roles of the Sirt1-p53 pathway, Akt-FOXO3a pathway and the p38 MAPK within the scope of MI-induced acceleration of cardiac remodeling in the diabetic heart. Sirtuin1 (Sirt1), a class III histone deacetylase (HDAC) has been shown to act as an endogenous inhibitor of apoptosis in cardiac myocytes [13] and Sirt1-mediated deacetylation of DNA-bound histones, p53 and forkhead class O 3a (FOXO3a) have been implicated in the inhibition of apoptosis and promotion of cellular growth [14], our previous study showed that Sirt1 is overexpressed in cardiomyocytes of diabetic Goto-Kakizaki rats [15]. The protein kinase B (PKB or Akt) has been identified as an important effector of the insulin/IGF - PI3K signaling pathway, acting as a regulator of cell survival and promoting cardiac hypertrophy [16-19]. FOXO3a transcription factor is negatively regulated by Akt, and in the absence of Akt-mediated phosphorylation FOXO3a has been shown to induce the expression of genes implicated in the apoptotic process [20]. Mitogen activated protein kinase (MAPK) p38 is activated after ischemic events and angiotensin II [21-23]. Long-term p38 MAPK activation has been shown to increase cardiac hypertrophy, fibrosis and apoptosis suggestive of a deleterious effect on the heart [24-26] and p38 MAPK overexpression has been shown to induce proinflammatory and profibrotic genes [27].

In the present study we examined whether the underlying diabetic pathology was exacerbated by experimental MI. In particular we wanted to investigate whether MI-induced cardiomyocyte hypertrophy, apoptosis and interstitial fibrosis was associated with changes in Akt - FOXO3a phosphorylation, Sirt1 - p53 deacetylation and p38 MAPK phosphorylation.

## Methods

### Myocardial infarction, blood pressure recordings and sample preparations

The investigation conforms to the *Guide for the care and use of laboratory animals *published by the US National Institutes of Health (NIH Publication No. 85-23, revised 1996). Experimental myocardial infarction was induced by ligating the left anterior descending (LAD) coronary artery in 23 eight-week old spontaneously diabetic Goto-Kakizaki rats (M&B, Denmark) (GK + MI) and 17 age-matched non-diabetic Wistar (M&B, Denmark) (W + MI) rats as described previously [28] under ketamine (50 mg/kg, i.p.) and medetomidine (10 mg/kg, i.p.) anesthesia. Long-acting insulin (1 IU/rat) was given two hours before anesthesia to GK rats to prevent hyperglycemia. The rats received postoperative pain reliever (buprenorphine 0.01-0.05 mg/kg s.c.) twice a day for two consecutive days. Sham-operated Goto-Kakizaki rats (GK) (n = 8) and Wistar rats (W) (n = 9) served as controls. 24 hours after surgery 8 GK rats were alive (GK + MI) and 10 Wistar rats were alive (W + MI). Systolic blood pressure was measured using a tail cuff blood pressure analyzer (Apollo-2AB Blood Pressure Analyzer, Model 179-2AB, IITC Life Science, Woodland Hills, CA, USA) at week 12. Urine samples were collected over a 24 h period in metabolic cages for catecholamine measurements using 1 M HCl as a preservative. Rats were anesthetized with CO_2_/O_2 _(AGA, Riihimäki, Finland) and decapitated. Blood samples were collected for biochemical measurements using EDTA as an anticoagulant. The hearts were excised, washed with ice-cold saline, blotted dry, weighed, and snap-frozen in liquid nitrogen or isopentane (-35°C). All samples were stored at -80°C until assayed.

### Echocardiography

Transthoracic echocardiography (Toshiba Ultrasound, Japan) was performed on all rats under isoflurane anesthesia (AGA, Riihimäki, Finland) in a blinded fashion by the same technician during the last study week. Each animal underwent three separate measurements by detaching the transducer between each measurement, and three pictures were taken. Parameters needed for the calculation of cardiac function and cardiac dimensions were measured from three systole-diastole cycles. By a two-dimensional imaging method (Teichholz method), using a 15-MHz linear transducer a short axis view of the left ventricle at the level of the papillary muscles was obtained. Two-dimensionally guided M-mode recording through the anterior and posterior walls of the left ventricle let us measure the left ventricle (LV) end-systolic (LVESD) and end-diastolic (LVEDD) dimensions as well as interventricular septum (IVS), anterior wall (AW) and posterior wall (PW) thickness. LV fractional shortening (FS) and ejection fraction (EF), measures of LV systolic function, were calculated from the M-mode LV dimensions using the following equations:

where LVEDV is the LV end-diastolic volume, equal to (7 × LVEDD^3^)/(2.4+(LVEDD) and LVESV is the LV end-systolic volume, equal to (7 × LVEDS^3^)/(2.4+(LVEDS). Cardiac output (CO) was calculated as a product of heart rate (HR) and stroke volume (SV = LVEDV-LVESV). CO (ml/min) = HR × SV.

### Infarct size, collagen volume fraction and cardiomyocyte cross sectional area

For histological analysis the hearts were fixed in 10% buffered formalin solution. Transversal sections of the left ventricle were embedded in paraffin and 5 μm-thick sections were cut and stained with Masson's trichrome or picrosirius red. The infarct sizes were determined planimetrically from the trichrome-stained histological sections as described previously [29]. In brief, the presence of collagen scars compatible with an old infarction was analyzed by examination of transverse LV sections. The size of MI was determined planimetrically as the ratio of infarct tissue or scar to the length of the entire LV endocardial circumference. Interstitial collagen volume fraction from the remote area was measured from picrosirius red-stained histological sections by light microscopy (×200) using computerized software (ImageJ, NIH). Conventional light microscopy at ×400 magnification was used to determine cardiomyocyte cross sectional area. 9-12 random fields from the remote area spanning the left ventricular septal wall were studied, and in each field the cell borders were measured from myocytes cut in short axis with a visible nucleus. An average of 60 cardiomyocytes per animal (n = 6) from each study group were studied. Cross sectional area was evaluated in a blinded fashion and analyzed using the ISI imaging software (Image Solutions Inc., Whippany NJ, USA).

### Immunohistochemistry

For immunohistochemistry, formalin-fixed myocardial sections were deparaffinized and rehydrated as described in detail previously [30]. Primary polyclonal antibody against Sirt1 (Sir2alpha, Rabbit polyclonal, Upstate; 1:200 dilution) and secondary biotinylated anti-rabbit antibody (Vector Laboratories Inc., California, USA) were used. The average percentages of Sirt1-positive cardiomyocytes/total number of cardiomyocytes were calculated from 6 separate fields of transverse left ventricular tissue sections using light microscopy (×400 magnification).

### Biochemical Analyses

Blood glucose was determined with a handheld test meter (Glucocard II^®^, Arkray, Japan), plasma BNP (BNP-45, Peninsula Laboratories, Belmont, CA, USA), plasma renin activity (Angiotensin I RIA kit, Diasorin, Italy), serum aldosterone (Coat-a-Count Aldosterone RIA kit, DPC Biermann, Bad Nauheim, Germany) and serum insulin (Rat insulin RIA kit, Linco, St. Charles, Missouri, USA) were determined by radioimmunoassay according to the instructions of the manufacturers. Urinary noradrenalin was analyzed using the isocratic ion-pair reversed-phase high-performance liquid chromatography method with electrochemical detection [31].

### TUNEL Apoptosis Assay

Cardiomyocyte apoptosis was assessed by the terminal deoxynucleotide transferase mediated ddUTP nick end labeling (TUNEL) assay as previously described [32]. In brief, apoptotic nuclear DNA strand breaks were end-labeled with digoxigenin-conjugated dideoxy-UTP by terminal transferase and visualized immunohistochemically with digoxigenin antibody conjugated to alkaline phosphatase. The assay was standardized with the use of adjacent tissue sections treated with DNase I to induce DNA fragmentation as a positive control for apoptosis. The proportion of TUNEL-positive cardiomyocytes was calculated from transverse left ventricular tissue sections using light microscopy (×200 magnification) with an ocular grid. Cardiomyocyte origin of the apoptotic cells was identified by the presence of myofilaments surrounding the nucleus. The proportion of apoptotic cardiomyocytes was counted in the border zones of infarct scars and in the remote non-infarct myocardium [28].

### Immunoblotting

Myocardial samples from left ventricles of MI - and Sham-operated GK and Wistar rats were electrophoretically separated by SDS-PAGE (20 μg total protein per lane). Proteins were transferred to a PVDF membrane (Immobilon-PR, Millipore, Bedford, MA, USA) and blocked in 5% non-fat milk-TBS - 0.01% Tween-20R buffer. The membranes were probed with the following antibodies: anti-caspase-3 (Upstate-Millipore), anti-Bax (Abcam) anti-phospho-FKHRL/FOXO3a (Cell Signaling Technologies), anti-FKHRL/FOXO3a (CST), anti-Akt (CST), anti-phospho-Akt (CST), anti-p38 (CST) and anti phospho-p38 (CST). Tubulin was used as loading control (Anti-alpha tubulin, Abcam, Cambridge, UK). Horseradish peroxidase-conjugated anti-rabbit secondary antibody (Chemicon) was subjected to enhanced chemiluminescence solution (ECL plus, Amersham Biosciences, Buckinghamshire, UK) and exposed to x-ray film (Hyperfilm-ECL, Amersham). We quantified the relative protein expression in separate samples from the x-ray film by densitometry (Synoptics, Cambridge, UK).

### Electrophoretic Mobility Shift Assay of Nuclear FOXO3a Transcription Factor

Electrophoretic mobility shift assay (EMSA) was used to determine DNA-binding of nuclear FOXO3a transcription factor. Nuclear proteins were extracted by grinding left ventricle samples from the remote area in liquid nitrogen and incubating in a buffer with protease inhibitor (Complete^®^, Roche), membrane proteins were solubilized with Nonidet P-40 (Roche Diagnostics GmBH, Mannheim, Germany). 5 μg of nuclear extracts were incubated in binding buffer (3 mM Tris pH 7.5, 50 mM NaCl, 5% Glycerol, 1 mmol/l EDTA, 0.01 mg/ml Poly [d(I-C)], 1 mmol/l DTT, 2 mg/ml BSA) and 1.25 fmol of digoxigenin-tagged FOXO3a oligonucleotides: 5'-DIG-ATT GCT AGC AAG CAA AAC AAA CCG CTA GCT TA-3' and 5'-DIG-TAA GCT AGC GGT TTG TTT TGC TTG CTA GCA AT-3' (Oligomer, Helsinki, Finland). After 30' incubation, the samples were electrophoretically separated by 5% SDS-PAGE and blotted to a nylon membrane (Hoefer semiPhor, TE77, Amersham Biosciences). Nuclear proteins were cross linked to the membrane for 10' in 254 nm UV-light. The membrane was probed with rabbit Anti-digoxigenin-AP-conjugate (Roche) for 30 min. and incubated in detection reagent (CDP Star, Roche) and exposed to an x-ray film (Kodak Inc., Japan). The indication reaction was done by incubating the samples with tagged and untagged oligonucleotides and probing with rabbit anti-FKHRL1 (sc-11351, Santa Cruz, CA, USA).

### Quantitative real-time RT-PCR

Total RNA from the rat hearts were extracted with Trizol^® ^(Gibco, Invitrogen, Carlsbad, CA, USA), treated with DNAse 1 (Deoxyribonuclease 1, Sigma Chemicals Co., St Louis, MO, USA) and reverse transcribed to cDNA by reverse transcription enzyme (Enhanced avian HS RT-PCR kit, Sigma Chemicals Co, Saint Louis, MO, USA). One μl of cDNA was subjected to quantitative real time polymerase chain reaction using the Lightcycler^® ^instrument (Roche diagnostics, Neuilly sur Seine, France) for detection of atrial natriuretic peptide (ANP), connective tissue growth factor (CTGF), interleukin-6 (IL-6) and ribosomal 18S mRNA. The following primers were used: ANP forward CCGATAGATTCTGCCCTCTTGAA, reverse CCCGAAGCAGCTTGATCTTC; CTGF forward GGCAGGGCCAACCACTGTGC, reverse CAGTGCACTTGCCTGGATGG; IL-6 forward AGGAAGGCAGTGTCACTCATTGT, reverse CTTGGGTCCTCATCCTGGAA and 18S forward ACATCCAAGGAAGGCAGCAG reverse TTTTCGTCACTACCTCCCCG. The samples were amplified using FastStart DNA Master SYBR Green 1 (Roche diagnostics) according to the protocol of the manufacturer.

### Statistical analyses

Data are presented as means ± SEM. Statistically significant differences in mean values were tested by analysis of variance (ANOVA) and the Neuman-Keul's post-hoc test for comparisons of multiple groups.

## Results

### Post-infarct heart failure in spontaneously diabetic Goto-Kakizaki rats

**Figure 1:**
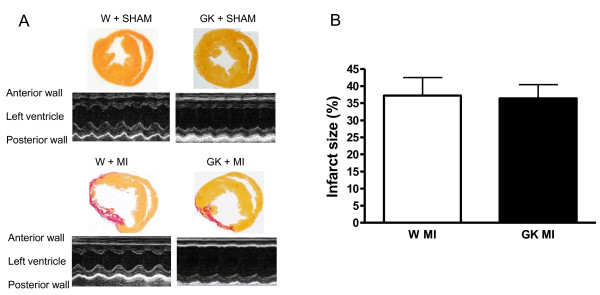
**Effect of myocardial infarction (MI) in diabetic Goto-Kakizaki (GK) rats and Wistar (W) rats 12 weeks after MI or Sham--operation**. (A) shows picrosirius--red stained whole heart section images (upper panels) and 600 ms from representative M-mode echocardiography (lower panels), (B) shows infarction area measured by planimetry from picrosirius--stained slides. Data is presented as means ± SEM.

24-hour survival after MI was 35% in Goto-Kakizaki rats and 55% in Wistar rats. One rat (in the Wistar + MI group) died during the follow-up period. At the end of the 12-week follow-up there were no differences in myocardial infarct size between GK + MI or Wistar + MI rats as assessed by planimetry (Figure 1). In both animal strains myocardial infarction led to thinning of the left ventricular anterior wall and systolic dysfunction as shown by decreased ejection fraction and fractional shortening (Table 1). MI reduced systolic blood pressure by 10 mmHg in Wistar and 19 mmHg in GK rats, systolic blood pressure was slightly higher in sham-operated GK rats (by 5 mmHg) (Table 1). All GK rats were hyperglycemic and GK + MI rats showed increased serum insulin concentration, all basic biochemical parameters are given in table 2.

**Table 1:** Systolic blood pressure and echocardiography data in diabetic Goto-Kakizaki (GK) rats and non-diabetic Wistar (W) rats 12 weeks post myocardial infarction (MI) or Sham operation, (d) denotes diastole and (s) systole.

	W Sham (n = 9)	GK Sham (n = 8)	W + MI (n = 9)	GK + MI (n = 8)
**Systolic blood pressure (mmHg)**	144 ± 7.9	149 ± 6.2	135 ± 5.4* ^#^	131 ± 14* ^#^
**Anterior wall thickness (d), (mm)**	1.8 ± 0.2	1.8 ± 0.3	1.3 ± 0.6	1.5 ± 0.7
**Anterior wall thickness (s), (mm)**	3.1 ± 0.1	3.1 ± 0.1	1.7 ± 0.4* ^#^	1.9 ± 0.4* ^#^
**LV inner diameter (d), (mm)**	8.6 ± 0.5	7.9 ± 0.6	10.4 ± 1.1* ^#^	10.0 ± 1.7^#^
**LV inner diameter (s), (mm)**	5.0 ± 0.2	4.4 ± 0.3	8.5 ± 0.7* ^#^	8.3 ± 0.8* ^#^
**Posterior wall thickness (d), (mm)**	1.9 ± 0.2	2.1 ± 0.2	2.3 ± 0.4	2.1 ± 0.6
**Posterior wall thickness (s), (mm)**	3.1 ± 0.1	3.2 ± 0.1	2.9 ± 0.2	2.5 ± 0.2
**End-diastolic volume, (ml)**	1.4 ± 0.2	1.1 ± 0.2	2.3 ± 0.6* ^#^	2.2 ± 0.9* ^#^
**End-systolic volume, (ml)**	0.3 ± 0.03	0.2 ± 0.03	1.52 ± 0.27* ^#^	1.4 ± 0.3* ^#^
**Heart rate, (min ^-1^)**	341 ± 26	360 ± 29	346 ± 19	302 ± 60^#^
**Ejection fraction (%)**	77.9 ± 1.9	80.5 ± 2.3	39.7 ± 7.7* ^#^	41.6 ± 6.9* ^#^
**Fractional shortening (%)**	42.3 ± 1.7	45.3 ± 2.7	18.9 ± 4.2* ^#^	19.1 ± 3.9* ^#^
**Cardiac Output (ml/min)**	368 ± 18.8	321 ± 23.7	258 ± 39.6*	228 ± 21.9*

The data is presented as means ± SEM.

* denotes *P *< 0.05 vs. W Sham, ^#^*P *< 0.05 vs. GK Sham, ^†^denotes *P *< 0.05 vs. W + MI.

**Table 2:** Basic biochemical parameters 12 weeks after MI- or sham operation in spontaneously diabetic Goto-Kakizaki (GK) and non-diabetic Wistar (W) rats.

	W Sham (n = 9)	GK Sham (n = 8)	W + MI (n = 9)	GK + MI (n = 8)
**B-Glucose (mmol/l)**	4.9 ± 0.24	8.2 ± 0.78*	4.2 ± 0.67	8.2 ± 0.10* ^†^
**S-Insulin (pg/ml)**	1.7 ± 0.58	2.2 ± 0.19	1.2 ± 0.24	3.3 ± 0.10* ^†^
**P-Renin activity (ng/ml/h)**	2.5 ± 0.24	2.1 ± 0.43	3.6 ± 0.43	1.9 ± 0.48^†^
**S-Aldosterone (pg/ml)**	91.8 ± 17.6	194 ± 27.3	171 ± 52.2	262 ± 23.3*
**U-Noradrenaline (nmol/24 h)**	4.5 ± 0.46	3.0 ± 0.27	6.0 ± 0.38^#^	4.5 ± 0.39^†^

The data are presented as means ± SEM.

* denotes *P *< 0.05 vs. W Sham, ^#^*P *< 0.05 vs. GK Sham and ^†^*P *< 0.05 vs. W + MI.

### Exacerbation of cardiomyocyte hypertrophy after MI in diabetic Goto-Kakizaki rats

**Figure 2:**
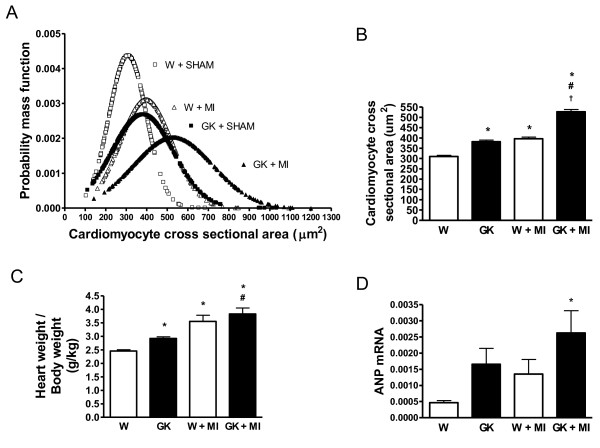
**Left ventricular hypertrophy is accentuated in the spontaneously diabetic Goto-Kakizaki (GK) rat after MI**. In (A) the normal distribution curves are shown of cardiomyocyte cross sectional areas (μm^2^) calculated from an average of 400 cardiomyocytes in each group (N = 6 per group). The mean values, presented as μm^2 ^of cardiomyocyte cross sectional areas, calculated from the total number of myocytes are presented in (B). Panel (C) shows the heart weight/body weight ratios (g/kg) and panel (D) shows the ANP mRNA expression as determined by qRT-PCR form the LV:s of GK and Wistar rats (N = 6). Data is presented as means ± SEM; * indicates *P *< 0.05 vs. W (sham-operated Wistar), ^# ^indicates *P *< 0.05 vs. GK (sham-operated GK), ^† ^indicates *P *< 0.05 vs. W+MI.

MI induced an increase in average cardiomyocyte size (1.4-fold in GK) as evaluated from cross sections of left ventricular tissue (Figure 2). Although MI also increased cardiomyocyte cross sectional area in non-diabetic Wistar rats, the average cardiomyocyte size in post-MI Wistar rats was comparable to sham-operated GK rats (Figure 2B). MI tended to increase ANP mRNA expression in Wistar and GK rats (Figure 2D).

### Sustained cardiomyocyte apoptosis and increased interstitial fibrosis after MI in the diabetic Goto-Kakizaki rat heart

**Figure 3:**
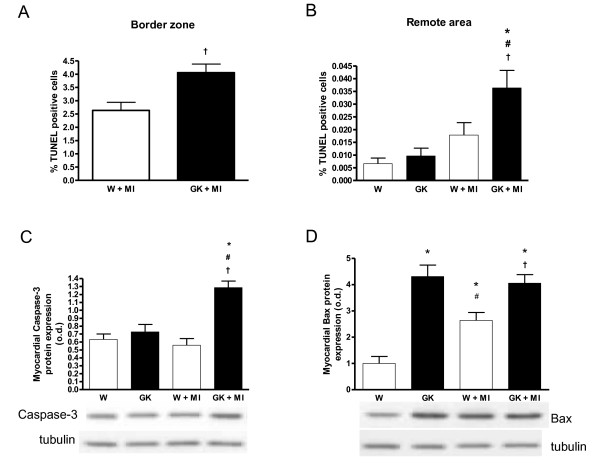
**Sustained cardiomyocyte apoptosis in diabetic GK rats 12 weeks after MI**. Results from TUNEL staining show increased cardiomyocyte apoptosis in the infarct border zone (A) and in the remote area (B) in GK rats (n = 6) 12 weeks after MI. Panel (C) shows myocardial caspase-3 protein overexpression in post--MI GK rats as assessed by western blotting. In panel (D), results from western blot shows an increase in proapoptotic Bax protein in sham- and MI operated GK rats and Wistar MI rats. The lower panels in (C) and (D) show representative western blots of myocardial tissue probed with anti-caspase-3, anti-bax and anti-tubulin. Data is presented as means ± SEM; * indicates *P *< 0.05 vs. W (sham-operated Wistar), ^# ^indicates *P *< 0.05 vs. GK (sham-operated GK), ^† ^indicates *P *< 0.05 vs. W+MI.

**Figure 4:**
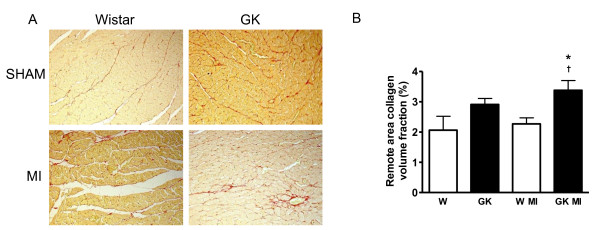
**Increased interstitial fibrosis in the remote area of diabetic GK rat myocardium 12 weeks after MI**. Panel (A) shows picrosirius--red stained photomicrograph images (×100 original magnitude), from the remote area of the left ventricle in sham- and MI operated GK and Wistar rats. In panel B, results from collagen volume fraction measurements show increased interstitial fibrosis in the remote area in GK rats with MI. Data is presented as means ± SEM; * indicates *P *< 0.05 vs. W (sham-operated Wistar, ^† ^indicates *P *< 0.05 vs. W+MI.

Post-infarct cardiomyocyte apoptosis was assessed by TUNEL staining. In agreement with previous studies [28], cardiomyocyte apoptosis was >10-fold higher in the border zone compared to the non-infarct remote area after MI both in diabetic and non-diabetic rats (Figure 3). TUNEL analysis showed that the number of apoptotic nuclei, measured from the border zone and in the area remote to the infarct, was higher in GK rats after MI as compared to Wistar controls (Figure 3). Sham-operated GK rat cardiomyocytes did not show overt apoptosis. Western blots revealed increased myocardial caspase-3 protein expression in GK rats after MI. Increased Bax protein expression was noted in both sham- and MI-operated GK rats, in Wistar however, MI induced an increase in Bax protein expression (Figure 3D). Interstitial collagen volume fraction measured from the remote area was increased in GK rats with MI compared to non-diabetic Wistar rats with MI (Figure 4).

### Akt - FOXO3a-dephosphorylation and FOXO3a-activation in diabetic Goto-Kakizaki rats after MI

**Figure 5:**
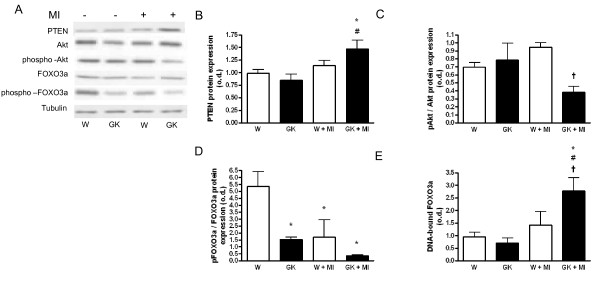
**Decreased Akt phosphorylation in diabetic GK rat myocardium and increased forkhead class O 3a (FOXO3a) DNA binding 12 weeks after MI**. Panel (A) shows representative western blots of myocardial tissue probed with anti-PTEN, anti-Akt, anti--phospho-Akt, anti-FOXO3a, anti-phospho-FOXO3a and anti-Tubulin as loading control. In (B) PTEN protein is overexpressed in GK+MI rats, (C) shows a decrease in the phosphorylated Akt/total Akt protein expression status in GK+MI rats compared to W+MI. In (D) phosphorylated FOXO3a/total FOXO3a protein expression status is shown. Panel (E) shows an increase in DNA--bound, myocardial FOXO3a transcription factor in the post-MI GK rats as assessed by electrophoretic mobility shift assay (EMSA). Data is presented as means ± SEM; * indicates *P *< 0.05 vs. W (sham-operated Wistar), ^# ^indicates *P *< 0.05 vs. GK (sham-operated GK), ^† ^indicates *P *< 0.05 vs. W+MI.

Our results showed that MI increased myocardial PTEN protein expression in GK but not in Wistar rats (Figure 5A). Phosphorylation of Akt kinase was decreased in GK rat hearts 12 weeks post MI compared to post-MI Wistar rats (Figure 5B). The phosphorylation status of Akt-regulated FOXO3a transcription factor was decreased in sham- and MI GK rats and MI Wistar rats (Figure 5C). Furthermore, the examination of DNA-bound FOXO3a by electrophoretic mobility-shift assay demonstrated a marked increase in nuclear, DNA bound FOXO3a in GK rats with MI compared to Sham-GK and corresponding post-MI Wistar controls (Figure 5D).

### Sirt1 Protein Overexpression and p53-Deacetylation in the Goto-Kakizaki rat Heart after MI

**Figure 6:**
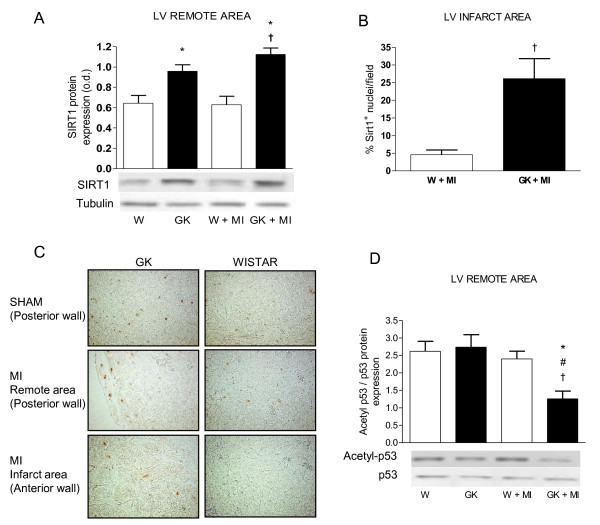
**Sirt1 protein overexpression and p53 deacetylation in diabetic GK rat myocardium 12 weeks after MI**. In (A) results from western blotting shows an increase in Sirt1-protein expression in the myocardium of GK rats 12 weeks after sham or MI operation. Results from immunohistochemistry show an increase in the number of Sirt1--positive nuclei from myocardial tissue sections in the infarct border zone (B). Panel (C) shows representative Sirt1-immunohistochemistry micrographs (×200) from posterior wall (sham and MI) and anterior wall (MI). Panel (D) shows a decrease in the acetylation of the Sirt1-target protein p53 in the myocardium of post-MI GK rats. The lower panels in (A) and (D) show representative western blots of myocardial tissue probed with anti-Sirt1, anti-tubulin, anti-p53 and anti-acetyl p53. Data is presented as means ± SEM; * indicates *P *< 0.05 vs. W (sham-operated Wistar), ^# ^indicates *P *< 0.05 vs. GK (sham-operated GK), ^† ^indicates *P *< 0.05 vs. W+MI.

We examined myocardial Sirt1-, acetyl-p53- and p53 protein expression by western blotting and Sirt1-localization by immunohistochemistry. Western blotting showed an increase in Sirt1 protein expression in sham-operated GK rats compared to Wistar with no further increase in Sirt1 protein expression after MI (Figure 6A). Immunohistochemical analysis revealed an increase in the number of Sirt1-positive nuclei in the infarct area of GK rats (Figure 6B). Acetylation of p53 at the Sirt1-preferred site was decreased in the LV remote area in post-MI GK rats compared to GK sham and post-MI Wistar rats (Figure 6D) as assessed by western blotting.

### Increased p38 MAP kinase phosphorylation in diabetic GK rats after MI

**Figure 7:**
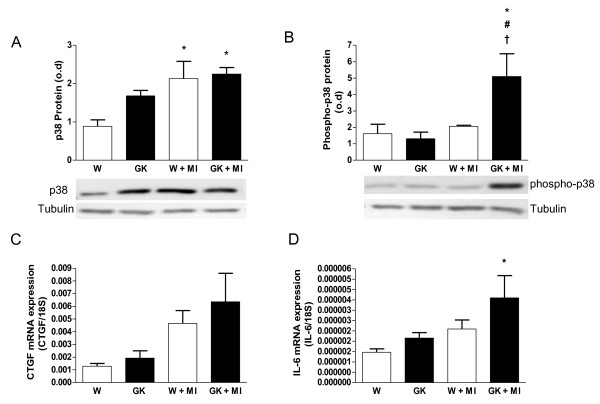
**Increased p38-phosphorylation in diabetic GK rats 12 weeks after MI**. Panel (A) shows an increase in total myocardial p38 protein expression in post-MI GK and Wistar rats. Panel (B) shows an increase in phosphorylated p38 in GK rats with MI. The lower panels in (A) and (B) shows representative western blots of total--p38 and phospho-p38 respectively, anti-tubulin was used as loading control. Panel (C) shows the mRNA expression of connective tissue growth factor (CTGF) in myocardial tissue and (D) shows the mRNA expression of interleukin -6 (IL-6) in myocardial tissue. Data is presented as means ± SEM; * indicates *P *< 0.05 vs. W (sham-operated Wistar), ^# ^indicates *P *< 0.05 vs. GK (sham-operated GK), ^† ^indicates *P *< 0.05 vs. W+MI.

Protein expression of myocardial p38 was increased after MI in Wistar rats (Figure 7A). However, the phosphorylation of p38 MAPK was increased only in post-MI GK rats (Figure 7B). The mRNA expressions of p38-regulated, profibrotic connective tissue growth factor (CTGF) and proinflammatory interleukin -6 (IL-6) tended to be increased in post-MI GK rats (Figure 7C-D).

## Discussion and Conclusions

Underlying cardiomyopathy has been attributed to an increase in mortality and morbidity after myocardial infarction in patients with diabetes. This study was conducted to investigate the molecular mechanisms of post-infarct cardiac remodeling, specifically the regulators involved in cell survival and apoptosis. Our study showed that diabetic GK rats were less likely to survive occlusion of the left ascending coronary artery than the control non-diabetic Wistar rats. The remaining animals survived the follow-up period, and at the 12-week cut-off point significant remodeling in the remaining, viable tissue of the heart had occurred. At 12 weeks post-MI, there were no significant differences in echocardiography parameters indicating that the samples were taken before decompensated heart failure. Earlier studies have shown that GK rats develop spontaneous impairment in systolic function [15] whereas others have shown the absence of overt systolic dysfunction at baseline in diabetic GK rats compared to Wistar [33]. Further acceleration in heart failure progression has been shown to occur 8 weeks postligation in GK rats [33]. Our study showed that baseline systolic function was similar between the Wistar and GK strains, and that MI induced a similar decrease in systolic function in diabetic and non-diabetic rats at the 12-week follow-up point. In diabetic rats there was however an ongoing exacerbation of post-infarct remodeling, such as increased cardiomyocyte hypertrophy, a sustained increase in cardiomyocyte apoptosis both at the remote and border zone of the infarct, and a slight increase in collagen deposition, evident 12 weeks postligation. The post-MI changes in non-diabetic rats included an increase in cardiac hypertrophy, an increase in cardiomyocyte apoptosis only at the border zone, and no changes in myocardial fibrosis. These macroscopic differences between the animal strains were associated with pronounced molecular changes in stress response pathways involved in cardiomyocyte hypertrophy, apoptosis and interstitial fibrosis in diabetic rats. Key changes in the posttranslational modification and expression of stress response proteins were decreased Akt phosphorylation and a concomitant increase in FOXO3a activation. Secondly, our results showed that Sirt1 protein was overexpressed in the myocardial tissue of GK rats and that one of the key Sirt1- targets; p53 was deacetylated in the diabetic heart compared to the non-diabetic situation. In the present study our results further showed that another Sirt1- target, FOXO3a was dephosphorylated, possibly due to Akt-kinase dephosphorylation in the diabetic GK rat heart. Thirdly, p38 MAPK protein phosphorlyation was increased in GK rats postligation.

Myocardial infarction leads to long-term functional changes in the left ventricle by altering the topography of infracted as well as non-infarct regions [34,35]. Infarct scarification and ventricular dilatation produces a mixture of pressure and volume overload in the non-infarcted region of the left ventricle [36]. The long-term effects of this pathological state includes ventricular remodeling where individual cardiomyocytes undergo maladaptive growth, ultimately leading to apoptosis, or adaptive growth which leads to compensatory hypertrophy of the ventricle [37]. After MI, exacerbation of hypertrophy occurs as a compensatory mechanism of remaining, functional left ventricular tissue to restore cardiac output [38]. In the present study, MI exacerbated hypertrophy in both Wistar and GK rats. As with our previous studies [15], pronounced cardiac hypertrophy was found in non-infarct GK rats compared to Wistar, which might be explained partly by the modest increase (+5 mmHg) in systemic blood pressure. Left ventricular hypertrophy was associated with an increase in ANP expression and low plasma renin activity, indicative of a volume overload in post-infarct GK rats, a feature that was not present in non-diabetic Wistar rats.

The Akt kinase pathway is activated by various stressful stimuli such as ischemia, heat, angiotensin II and circulating insulin. A previous study revealed that essential components of the insulin signaling pathway upstream of the Akt kinase, such as insulin receptor β, IRS-1 and GLUT4 protein, were impaired in the GK rat heart [39] which, at least in theory could affect the ability of the GK rat heart to respond to stress adequately. The phosphoinositide 3'-phosphatase [phosphoinositide and tensin homologue on chromosome 10 (PTEN)], dephosphorylates mediators of the PI3K signaling pathway, acting as a negative regulator of the anti-apoptotic effects of Akt [40-42]. In the present study we found that MI did not increase Akt phosphorylation in GK rats. PTEN phosphatase expression was slightly increased after MI in GK rats which might explain the decrease in Akt kinase phosphorylation level. Although Akt phosphorylation was decreased, GK rats showed pronounced increase in cardiomyocyte hypertrophy suggesting the involvement of non-Akt pathways in the development of diabetes-induced cardiac hypertrophy in this model.

FOXO3a is one of the effectors through which Akt suppresses apoptosis. FOXO3a-phosphorylation by Akt results in cytoplasm translocation and ubiquitination and subsequent down regulation of FOXO3a- target genes [43]. In contrast unphosphorylated FOXO3a is transported in response to stress into the nucleus where it induces the transcription of FOXO3a-regulated target genes. Nuclear FOXO3a favors expression of genes regulating the proapoptotic program [20]. In the present study, we showed that decreased Akt phosphorylation in the post-infarct GK rat myocardium was associated with a decrease in the phosphorylation of FOXO3a at Akt-preferred Ser253 amino acid. The decrease in phosphorylation was associated with increased nuclear FOXO3a-translocation and DNA binding as assessed by electrophoretic mobility shift assay. These results were associated with an increase in cardiomyocyte apoptosis in the GK rat heart as assessed by TUNEL stain. In the remote area and the area next to the infarct the number of apoptotic cells was higher in GK rats. Our study showed further evidence of apoptotic post-infarct cardiac remodeling in GK rats with a marked increase in the protein expression of key regulators of apoptosis, namely the caspase -3 and Bax-proteins. Further we showed that collagen volume fraction was increased in the interstitial tissue of the GK rat myocardium.

Our study showed that Sirt1 protein was overexpressed in both the remote area (posterior wall) and in the infarct area (anterior wall). Western blotting from remote area confirmed the result from our earlier studies [15], that Sirt1 protein was overexpressed in GK rats. In contrast, Sirt1 protein in post-infarct Wistar rats was not readily locatable in the infarct area. Furthermore we showed that increased Sirt1 protein in the remote area was associated with deacetylation of the Sirt1-target p53 in GK + MI rats. Sirt1 overexpression has been shown to protect cardiomyocytes from apoptosis, increase cellular stress resistance and cause modest hypertrophy [13,44] suggesting that an increase in Sirt1 protein expression during heart failure may be cardio protective. Decreased Sirt1- mediated p53-acetylation has been shown to be associated with a decrease in p53-mediated apoptosis. According to our results, however apoptosis was increased in the GK + MI rat heart in spite of the increased Sirt1-p53 deacetylation, although modestly with only 0.20% TUNEL-positive cells in the border zone of GK + MI rats. The increase in caspase-3 and Bax protein expression confirm these results. Taken together we suggest that Sirt1 may be involved in the post-MI remodeling process possibly protecting it from apoptosis but inducing hypertrophy.

In the present study we showed an increase in the phosphorylation of the p38 MAPK in the diabetic post-infarct GK rat myocardium. The p38 MAPK has been shown to be activated by hypoxia, inflammation, transforming growth factor beta and other growth factors as well as by angiotensin II [21-23]. The exact role of p38 in cardiac dysfunction is unclear. It has been suggested that long-term activation of p38 is deleterious and that p38 may accelerate cardiomyocyte growth during the transition from adaptive to maladaptive cardiac hypertrophy [24].

In the present study, the infarct exacerbated cardiac hypertrophy and fibrosis in the GK rat. While Akt kinase activation could not explain this effect, the increase in p38 MAP kinase phosphorylation might have played a role. Previous studies have indicated an increase in cardiac fibrosis in response to p38 MAPK overexpression and shown that adenoviral p38-injection into the heart increased CTGF mRNA expression [27]. We showed here that in the diabetic heart a higher level of cardiac collagen deposition was associated with an increase in p38 phosphorylation in myocardial tissue 12 weeks after MI. P38 MAPK activation has been associated with an increase in the expression of pro-inflammatory regulators such as interleukin-6 (IL-6), cyclooxygenase-2 (COX-2) and CCL2 in the heart [27]. In the present study we showed a tendency for IL-6 and CTGF mRNA expressions to be increased after MI in diabetic GK rats.

Together these results indicate that markers of stress response during the stable period of heart dysfunction are different in the diabetic versus non-diabetic heart. In GK rats, MI induced an increase in PTEN protein expression, reduced Akt phosphorylation and a concomitant decrease in FOXO3a phosphorylation which might have contributed to the increase in cardiomyocyte apoptosis seen here. We showed that Sirt1 protein overexpression is sustained after MI in diabetic rats, and that is localized to the nuclei at the infarct area. Sirt1 protein overexpression was further associated with a decrease in p53 acetylation in GK rats postligation. Furthermore our results support the notion that long-term p38 MAP-kinase activation may increase post-infarct cardiac remodeling in the diabetic GK rat heart, promoting an increase in fibrosis, cardiomyocyte hypertrophy and an increase in the expression of pro-inflammatory regulators. In conclusion post-infarct cardiac remodeling in diabetic GK rats included increased cardiomyocyte hypertrophy, increased cardiomyocyte apoptosis and interstitial fibrosis. The present study suggests an important role for Akt-FOXO3a, Sirt1-p53 and p38 in the regulation of post-infarct cardiac remodeling in type 2 diabetes.

## Competing interests

The authors declare that they have no competing interests.

## Authors' contributions

All authors have read and approved the final manuscript. EV performed immunohistochemistry and some of the western blot experiments and wrote the draft manuscript, ML performed the PCR experiments, HF and SM performed in vivo animal experiments (ligation, blood pressure etc.), JR performed the majority of the western blotting experiments, PK performed the echocardiography measurements, VK performed all TUNEL stainings, JL made all biochemical measurements (table 1), IT and EM proofread the manuscript.
